# Monochromatic Light Impacts the Growth Performance, Intestinal Morphology, Barrier Function, Antioxidant Status, and Microflora of Yangzhou Geese

**DOI:** 10.3390/ani15121815

**Published:** 2025-06-19

**Authors:** Gang Luo, Yiyi Cheng, Yingqing Xu, Jie Liu, Wen Yang, Jiying Liu, Binbin Guo, Huanxi Zhu

**Affiliations:** 1 Jiangsu Key Laboratory of Sericultural and Animal Biotechnology, School of Biotechnology, Jiangsu University of Science and Technology, Zhenjiang 212100, China; luo_gang1989@163.com (G.L.); 15036516192@163.com (Y.C.); xyingqing233@163.com (Y.X.); yangwen9901@163.com (W.Y.); liujiying@just.edu.cn (J.L.); 2 Key Laboratory of Silkworm and Mulberry Genetic Improvement, Ministry of Agriculture and Rural Affairs, Sericultural Scientific Research Center, Chinese Academy of Agricultural Sciences, Zhenjiang 212100, China; 3 Institute of Animal Science, Jiangsu Academy of Agricultural Sciences, Nanjing 210014, China; liujie891213@163.com; 4 Key Laboratory of Crop and Livestock Integration, Ministry of Agriculture, Nanjing 210014, China

**Keywords:** goose, growth performance, gut microflora, melatonin, monochromatic light

## Abstract

This study investigates the effect of monochromatic light on the body weight (BW), melatonin concentration and its receptors expression levels, intestinal health, and gut microorganisms of Yangzhou geese. Green light (GL) significantly increased BW, melatonin and its receptor expression levels, villus height (VH) and villus height/crypt depth (VH/CD) ratio, superoxide dismutase (SOD), catalase (CAT), and total antioxidant capacity (T-AOC) activities, as well as the abundance of *Synergistota* and *Prevotellaceae_UCG-001*, compared with white light (WL). Blue light (BL) significantly increased the mRNA expression of melatonin membrane receptor 1a (Mel1a) and nuclear receptor 1α (RORα), VH and VH/CD ratio, CAT activity, cecal microbes diversity, and decreased malondialdehyde (MDA) levels. Red light (RL) significantly decreased average daily feed intake, reduced the abundances of *Synergistota* and *Prevotellaceae_UCG-001*, and increased Mel1a and RORα mRNA expression levels, MDA content, and cecum microbial diversity. Moreover, melatonin levels were significantly higher in the GL and BL groups compared to RL. Furthermore, the mRNA expression levels of Claudin-10, Occludin, and occludens-1 (ZO-1) were significantly upregulated under GL or BL exposures compared to the WL group, whereas RL only enhanced the expression levels of ZO-1. Spearman’s correlation analysis revealed that the relative abundance of *Prevotellaceae_UCG-001* exhibited positive correlations with BW, melatonin and its receptors expression, gut health, and antioxidant capacity. Overall, these findings suggested that GL exposure enhanced melatonin synthesis and its receptors expression, modulated intestinal homeostasis and microbial ecology, and ultimately increased goose BW.

## Simple Summary

Monochromatic light impacts poultry growth, yet the underlying mechanisms, especially in highly light-sensitive geese, are not fully elucidated. In this study, Yangzhou geese were reared under white, green, red, or blue light for 70 days, and the feed intake, body weight, melatonin and its receptor expression level, intestinal morphology, barrier function, antioxidant status, and microflora were measured. The results indicated that the green light environment is more conducive to the growth of Yangzhou geese. Key indicators, including body weight and average daily feed intake (reflecting growth performance), villus height, villus height-to-crypt depth ratio, antioxidant indices, gut microbiota composition and abundance (reflecting intestinal health), and melatonin and its receptors (light signal “messenger”) were improved in the green light group in comparison with the other light groups. However, red light exerted the opposite effect, i.e., red light was unfavorable to the growth of Yangzhou geese. Based on the above observation, one might speculate that melatonin regulates gut morphology, intestinal microflora, and gut health through its receptors and then promotes growth performance in geese. This study presents the first investigation into the effects of monochromatic light exposure on the growth performance of Yangzhou geese through the gut–microbiome–brain axis.

## 1. Introduction

Environmental conditions, particularly light, can significantly influence the growth, reproduction, and behavior of animals [1,2,3]. Unlike mammals, birds have four-color vision, with optic cone cells in their eyes that perceive green, red, blue, and ultraviolet light. The spectral sensitivity of these cells covers green light (545–575 nm), orange/red light (580–700 nm), blue light (400–480 nm), and ultraviolet light (300–400 nm), with particular sensitivity to green light [4,5]. Stimulation with monochromatic green light reduces incubation time and enhances embryonic development by affecting pineal function and liver glucose metabolism [6,7,8]. Moreover, the use of monochromatic green or blue light enhances poultry growth performance. For instance, exposure to green light significantly increased the body weight of 70-day-old geese (*p* < 0.05), suggesting that environmental light color manipulation may serve as an effective strategy to enhance growth performance in waterfowl production [9]. However, the specific mechanisms through which green light influences geese growth remain unexplored.

Gut microbiota establish a symbiotic relationship with the host intestinal tract [10]. Current evidence indicates that the gut microbiota play a crucial role in regulating a range of host physiological functions, including digestion and absorption, immune response, and growth performance [11,12,13,14]. In poultry, the jejunal microbiota and cecal flora have been shown to enhance growth performance by mitigating intestinal inflammation and modulating lipid metabolism, respectively [14,15]. Moreover, light exposure affects the composition of the intestinal flora, with blue light specifically promoting the development of the small intestine and influencing intestinal function by reducing oxidative stress [16]. Therefore, exploring gut health and gut microbes is important for studying the light color modulation of poultry growth performance.

Melatonin is an important light-responsive messenger. It plays crucial roles in immune regulation and anti-oxidation [17,18]. Moreover, it mediates the effects of monochromatic light on chicken growth and development [19]. Geese are seasonal breeders that are particularly sensitive to light changes. Therefore, this study hypothesizes that monochromatic light influences the growth of geese by regulating intestinal function and flora, with melatonin potentially playing a direct or indirect role.

This study represents the inaugural investigation into the impact of monochromatic light exposure on the growth performance of Yangzhou geese via the gut–microbiome–brain axis. The results indicated that green light exposure significantly increased the body weight of Yangzhou geese, whereas exposure to red light exerted inhibitory effects on feeding behavior, as evidenced by a reduction in average daily feed intake. Notably, both intestinal melatonin receptors (Mel1a and RORα) and microbial diversity exhibited wavelength-dependent variations, with significant alterations observed across all three light spectra. Specifically, green light exposure significantly enriched the relative abundance of *Synergistota* and *Prevotellaceae_UCG-001*, while red light exposure resulted in a marked depletion of these bacterial populations. Moreover, *Prevotellaceae_UCG-001* demonstrated a positive correlation with growth performance, melatonin levels, and gut health. These spectral-specific alterations in neuroendocrine signaling and microbial community diversity suggest a novel photobiological mechanism that may influence the growth performance of Yangzhou geese through the gut–microbiome–brain axis. This study offers both mechanistic insights and practical applications for optimizing growth performance in Yangzhou geese through light management strategies.

## 2. Materials and Methods

### 2.1. Ethics Statement

All of the experiments and animal care procedures were approved by the Ethics Committee of the Jiangsu Academy of Agricultural Sciences and followed the rules set on 8 July 2014 (Decree No. 63).

### 2.2. Animals and Treatments

Male Yangzhou geese (1-day-old) were purchased from Jiangsu Guiliu Animal Husbandry Group Co., Ltd. (Xuzhou, China) and raised at the Poultry Farm of the Liuhe Animal Science Base of Jiangsu Academy of Agricultural Sciences in Jiangsu, China. In total, 240 goslings were randomly divided into four monochromatic light treatment groups: white light (WL, 400–700 nm), green light (GL, 560 nm), blue light (BL, 480 nm), and red light (RL, 660 nm). There were four replicates for each treatment and 15 birds for each replicate. The initial body weight (BW) of the four groups were 98.47 ± 1.82 g, 97.86 ± 0.35 g, 97.76 ± 1.19 g, and 97.35± 1.27 g, respectively, without differences between groups. All geese were housed in nets separated by light-sheltering materials to avoid crosstalk. Light intensity was maintained at 40 lx at the level of the geese head, and the light regime was 16 h light/8 h dark (lights off from 22:00 to 06:00). The light sources were calibrated every 10 d. From day 1 to day 20, the stocking density was 15 birds/m^2^, and was then reduced to 7.5 birds/m^2^ from day 21 to day 70. The room temperature was maintained at 28–30 °C by the hot water heating system for the first 20 days, and then gradually transitioned to room temperature until the end of the experiment. The manure was cleaned twice per day using an automatic conveyor system. The geese had free access to food and water.

### 2.3. Growth Performance

During the experiment, feed was recorded twice daily at 07:00 and 16:00. Before the first daily feeding, the amount of feed remaining in the trough was weighed and recorded for each replicate. The average daily feed intake (ADFI) was calculated by subtracting the weight of the remaining feed from the total feed supplied. On day 70, the BW of geese in each replicate was individually weighed and recorded. The feed conversion ratio (FCR) was defined as the ratio of feed consumed to body weight gain throughout the experiment.

### 2.4. Sample Collections

On day 70, 10 geese from each group were randomly selected and then slaughtered by decapitation after blood collection. The duodenum, jejunum, and ileum were harvested and washed with PBS. A part of the small intestine tissue was fixed with 4% paraformaldehyde for hematoxylin and eosin staining (H&E), and some jejunum tissues were collected and placed in cryopreservation tubes, transported by liquid nitrogen, and stored at −80 °C for qRT-PCR and antioxidant capacity analysis. Cecal contents were also collected for microbial sequencing and analysis.

### 2.5. Intestinal Morphology

Small intestinal tissues were fixed in 4% paraformaldehyde for 72 h. The sections were then thoroughly rinsed with PBS. Subsequently, tissues were dehydrated using a series of decreasing ethyl alcohol concentrations. Following complete dehydration, the tissues were rendered transparent using xylene. The final step involved immersing the tissues in wax to prepare 5 µm thick paraffin sections. The paraffin sections were dewaxed, dehydrated using xylene and a gradient of ethyl alcohol, and stained with H&E for histomorphological observation. Microscopic imaging was conducted using a light microscope (Olympus BX50, Tokyo, Japan) equipped with Olympus XC10 microdigital camera (Olympus Corporation, Tokyo, Japan). After 20-fold imaging with SlideViewer 2.5 software, the ImageJ Fiji 2.3.1 software was used for precise measurement of 10 complete villus heights (VH) and crypt depths (CD) per section (*n* = 6). The VH/CD ratio was calculated by dividing villus height by crypt depth.

### 2.6. Detection of Serum Melatonin Level

Blood samples per each group (*n* = 8) were collected from 70 d Yangzhou geese and centrifuged at 3000 rpm for 10 min. Serum was carefully extracted from the samples to measure melatonin levels using an enzyme-linked immunosorbent assay kit (Chejeter Technology Co., Ltd., Beijing, China). The intra- and inter-assay coefficients of variation were less than 10% and 15%, respectively.

### 2.7. Antioxidant Activity Assays

Geese jejunal tissues per each group (*n* = 6) were homogenized in 9-fold (m/v) cold PBS. After centrifugation at 3000 rpm for 10 min, the supernatant was collected for the detection of antioxidant activity. The antioxidant status of the jejunum was determined by measuring the activities of total superoxide dismutase (T-SOD), catalase (CAT), and total antioxidant capacity (T-AOC), as well as the content of malondialdehyde (MDA), according to the manufacturer’s instructions (Nanjing Jiancheng Bioengineering, Nanjing, China).

### 2.8. Quantitative Real-Time Polymerase Chain Reaction (qRT-PCR)

Total RNA (*n* = 6) was extracted using TRIzol reagent (R401-01, Vazyme, Nanjing, China), and the concentration and integrity of the extracted RNA were determined using a nucleic acid protein detector (IMPLEN, Munich, Germany). Subsequently, RNA was converted into cDNA by reverse transcription using a kit supplied by Takara (Cat# RR047A, Takara, Dalian, China). qRT-PCR amplification was performed using MagicSYBR Mixture (CW3008M, CWBIO, Beijing, China). Each experiment was repeated three times to ensure accuracy. Relative mRNA levels were normalized to the expression of glyceraldehyde-3-phosphate dehydrogenase (GAPDH), which served as an internal control. A comprehensive list of all primers used in this study is presented in Table 1.

### 2.9. Microbial Sequencing and Analysis

Total DNA was extracted from 32 cecal chyme samples using a HiPure Stool DNA Kit (Magen Biotechnology Co., Ltd., Guangzhou, China), according to the manufacturer’s instructions. The concentration was measured using a NanoDrop 2000 (Thermo Scientific, Waltham, MA, USA), and the quality of the DNA was detected by agarose gel electrophoresis. DNA samples were PCR-amplified by using barcoded primers flanking the V3–V4 region of the 16S rRNA gene. PCR was performed using Q5 @ High-Fidelity DNA polymerase. The amplification procedure was as follows: 95 °C for 5 min; followed by 30 cycles of 95 °C for 1 min, 60 °C for 1 min, and 72 °C for 1 min; and then 72 °C for 7 min. The PCR products were subjected to quality assessment by electrophoresis on a 2% agarose gel and subsequently purified using AMPure XP Beads (Beckman Coulter, Carlsbad, CA, USA; Catalog #B37419AB), according to the manufacturer’s standard procedure. Sequencing libraries were prepared using the Illumina DNA Prep Kit (Illumina, CA, USA), following the manufacturer’s protocol (https://support-docs.illumina.com/LP/IlluminaDNAPrep/Content/LP/Illumina_DNA/DNA-Prep/Protocol_IDP.htm (accessed on 16 December 2024). The libraries were subsequently sequenced on an Illumina sequencing platform by GeneDenovo Biotechnology Co., Ltd. (Guangzhou, China). The comprehensive analysis of the sequencing data, encompassing both α-diversity and β-diversity, was conducted on the Omicsmart platform, a service offered by Genedenovo Biotechnology Co., Ltd.

### 2.10. Statistical Analysis of Data

Data in graphs are presented as mean ± SEM. Differences between all data, except the sequencing data for gut microbes, were analyzed using one-way ANOVA followed by Duncan’s multiple range test for post hoc comparisons and plotted using GraphPad Prism 9 software (GraphPad Software, San Diego, CA, USA). For the assessment of α-diversity and the distribution of cecal microbiota at the phylum and genus levels, the Kruskal–Wallis test and the Wilcoxon Rank Sum Test were applied, respectively, focusing on comparisons between two of the four light color treatments. The evaluation of β-diversity was conducted utilizing the weighted UniFrac distance metric, and the findings were illustrated via principal coordinate analysis (PCoA). Correlation analyses of microflora with growth performance, melatonin levels, melatonin receptor expression, and gut health parameters were performed using the OmicSmart platform (https://www.omicsmart.com/). Statistical significance was set at *p* < 0.05.

## 3. Results

### 3.1. Effect of Monochromatic Light on Growth Performance

As shown in Table 2, GL exposure significantly enhanced the BW of Yangzhou geese at 70 days of age compared to the WL or RL groups (*p* < 0.05). Conversely, no significant variation in BW was observed among the WL, BL, and RL groups (*p* > 0.05). Moreover, the ADFI was notably lower (*p* < 0.05) in the RL group than in the WL, GL, and BL groups. Additionally, FCR remained unaffected by monochromatic light treatments compared to the WL group (*p* > 0.05). In summary, monochromatic light affects the growth performance of Yangzhou geese.

### 3.2. Effect of Monochromatic Light on Intestinal Morphology

**Figure 1:**
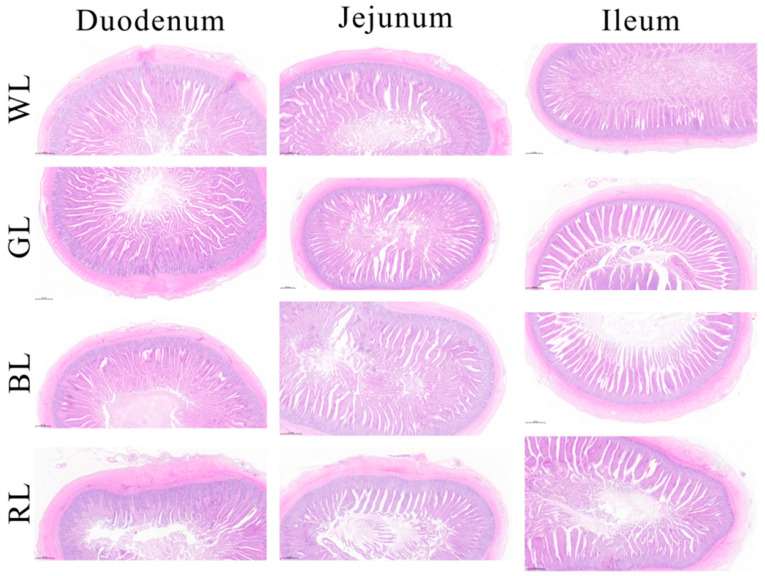
The intestinal (duodenum, jejunum, and ileum) morphology of Yangzhou geese under various monochromatic light. WL: White light; GL: Green light; BL: Blue light; RL: Red light. Scale bar: 500 μm.

As illustrated in Figure 1, morphological alterations in the duodenum, jejunum, and ileum were observed following exposure to different monochromatic light treatments. Compared to the WL group, the GL and BL treatments significantly enhanced the VH and the VH to CD ratio in the small intestine (*p* < 0.05) (Table 3), indicating improved intestinal digestion and absorption capacity [20]. Conversely, RL treatment led to a significant reduction (*p* < 0.05) in both the VH value and VH/CD ratios in the jejunum and ileum in comparison with other treatments, whereas no significant changes were observed in the duodenum (Table 3).

### 3.3. Serum Melatonin Levels and Intestine Melatonin Receptors Gene Expression

**Figure 2:**
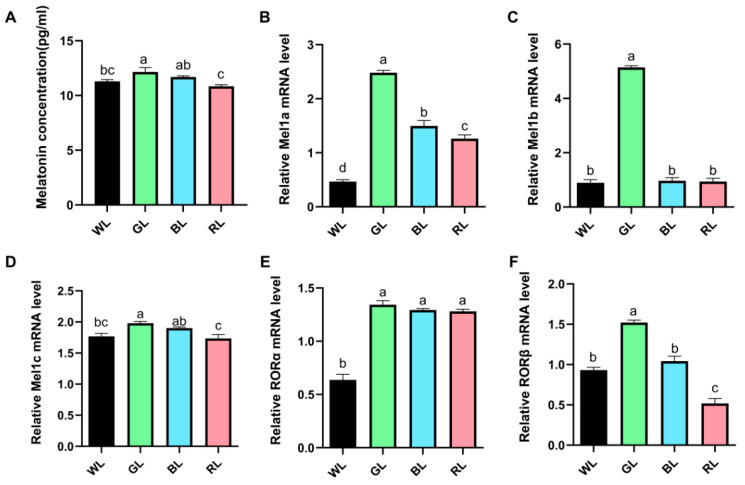
Effect of different monochromatic light on serum melatonin level and jejunum melatonin receptor mRNA expression. (**A**) Concentration of serum melatonin. (**B**–**D**) Relative mRNA expression of melatonin membrane receptor 1a, 1b, and 1c. (**E**,**F**) Relative mRNA expression of melatonin nuclear receptor RORα and RORβ. Data are expressed as means ± SEM. Significant differences (*p* < 0.05) among treatment groups are indicated by different superscript letters (a, b, c), while common letters represent non-significant differences. WL: White light; GL: Green light; BL: Blue light; RL: Red light. ^a,b,c^ Different superscript letters indicate statistically significant differences (*p* < 0.05), while common letters denote non-significant differences.

To further investigate the mechanisms underlying the light-regulated growth performance, serum melatonin levels and intestinal melatonin receptor expression were evaluated. As depicted in Figure 2A, GL significantly (*p* < 0.05) elevated serum melatonin levels compared to WL, whereas both GL and BL substantially enhanced (*p* < 0.05) serum melatonin levels relative to RL. Moreover, GL significantly (*p* < 0.05) upregulated the mRNA expression levels of melatonin membrane receptor (Mel) 1a, 1b, and 1c, as well as the nuclear receptors retinoic acid receptor-related orphan receptor α (RORα) and β (RORβ) in the jejunum, compared to WL (Figure 2B–F). Furthermore, BL or RL significantly (*p* < 0.05) enhanced the mRNA expression levels of Mel1a and RORα, whereas RL notably (*p* < 0.05) reduced the expression levels of RORβ (Figure 2).

### 3.4. Mucosal Barrier Functions

**Figure 3:**
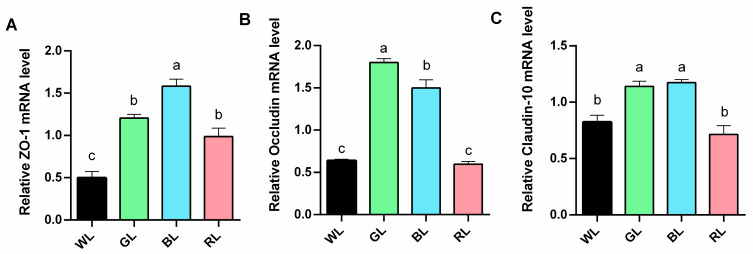
The impact of different monochromatic light on mucosal barrier. (**A**–**C**) Relative mRNA expression of ZO-1, occludin, and claudin-10. Data are expressed as means ± SEM (*n* = 6). Significant differences (*p* < 0.05) among treatment groups are indicated by different superscript letters (a, b, c), while common letters represent non-significant differences. WL: White light; GL: Green light; BL: Blue light; RL: Red light. ^a,b,c^ Different superscript letters indicate statistically significant differences (*p* < 0.05), while common letters denote non-significant differences.

Expression levels of tight junction proteins in the intestine were also analyzed (Figure 3). Compared to WL, GL and BL treatments significantly (*p* < 0.05) upregulated the mRNA expression of jejunal zonula occludens-1 (ZO-1), occludin, and claudin-10, whereas RL treatment significantly (*p* < 0.05) increased the mRNA expression of ZO-1 without exerting a significant effect on the expression of occludin and claudin-10.

### 3.5. Antioxidant Status of the Jejunum of Meat Geese

**Figure 4:**
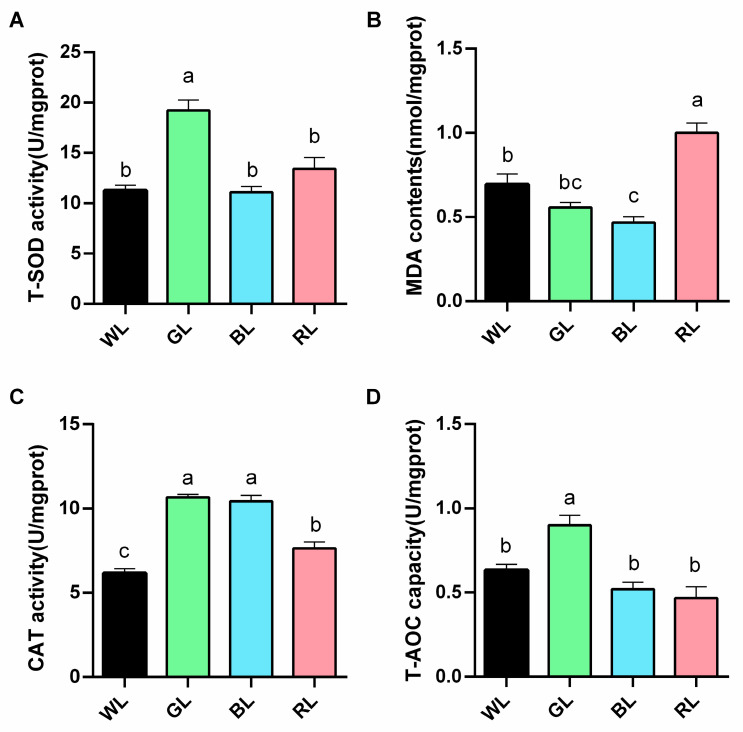
The effects of different monochromatic light on the antioxidant activity of the jejunum. (**A**) superoxide dismutase (T-SOD), (**B**) malonaldehyde (MDA), (**C**) catalase (CAT), and (**D**) total antioxidant capacity (T-AOC). Data are expressed as means ± SEM (*n* = 6). Significant differences (*p* < 0.05) among WL, GL, BL, and RL groups are indicated by different superscript letters (a, b, c), while common letters represent non-significant differences. WL: White light; GL: Green light; BL: Blue light; RL: Red light. ^a,b,c^ Different superscript letters indicate statistically significant differences (*p* < 0.05), while common letters denote non-significant differences.

In comparison with the WL group, GL treatment resulted in a significant (*p* < 0.05) enhancement of T-SOD, CAT, and T-AOC activities in the jejunum, while also demonstrating a tendency (*p* > 0.05) to reduce the MDA content. Similarly, BL treatment significantly increased CAT activity (*p* < 0.01) and reduced MDA levels (*p* < 0.05), whereas RL treatment significantly increased MDA levels (Figure 4).

### 3.6. Effect of Different Monochromatic Light on the Diversity of Intestinal Microflora

**Figure 5:**
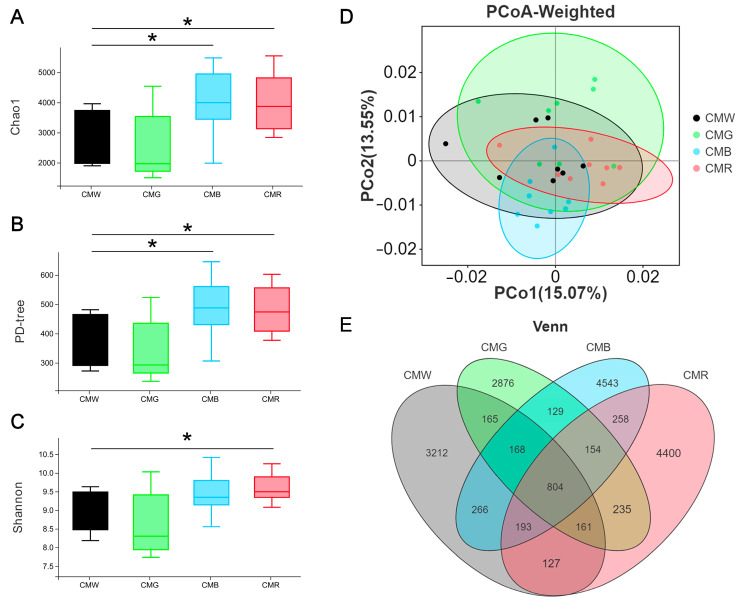
Impact of monochromatic light on microflora diversity and composition in cecum. (**A**–**C**) Boxplot illustrating inter-group differences in alpha diversity indices. (**D**) Principal coordinate analysis (PCoA) based on weighted UniFrac method. (**E**) Venn diagrams illustrating the distribution of operational taxonomic units (OTUs) in geese exposed to different monochromatic light. Student’s *t*-test was used to calculate *p*-values, defining *p* < 0.05 as statistically significant and marked with *. CMW, CMG, CMB, and CMR represent the cecal microbiota under white light, green light, blue light, and red light conditions, respectively.

Given the crucial role of gut microbes in animal growth, this study investigated the impact of monochromatic light on the gut microbial strains. In total, 91,235, 80,034, 106,964, and 99,786 raw reads were obtained from 32 samples of cecal contents across the four groups: WL (cecal microflora in the WL group, CMW), GL (CMG), BL (CMB), and RL (CMR), respectively. Correspondingly, the number of clean tags free from chimeras was 66,656, 61,327, 75,063, and 67,677 for each group, respectively. Regarding α-diversity, the Chao1 and PD-tree indices of the CMB and CMR groups were found to be significantly higher than those of CMW and CMG groups (Figure 5A,B). Additionally, the Shannon index was significantly higher in the CMR group than that in the CMW group (Figure 5C, *p* = 0.036446). In terms of β-diversity, PCoA based on weighted UniFrac distances in the cecum revealed significant differences in the microbial communities between the CMR and CMW groups (*p* = 0.00426). Compared to CMW, the number of operational taxonomic units (OTUs) decreased in CMG and increased in both CMB and CMR, with CMB exhibiting the highest number of OTUs (Figure 5E). The α-diversity and β-diversity indices indicated that the cecal microflora of CMW closely resembled that of CMG, while differing from that of CMR and CMB.

### 3.7. Changes in Gut Microbiota Composition at the Phylum and Genus Levels

**Figure 6:**
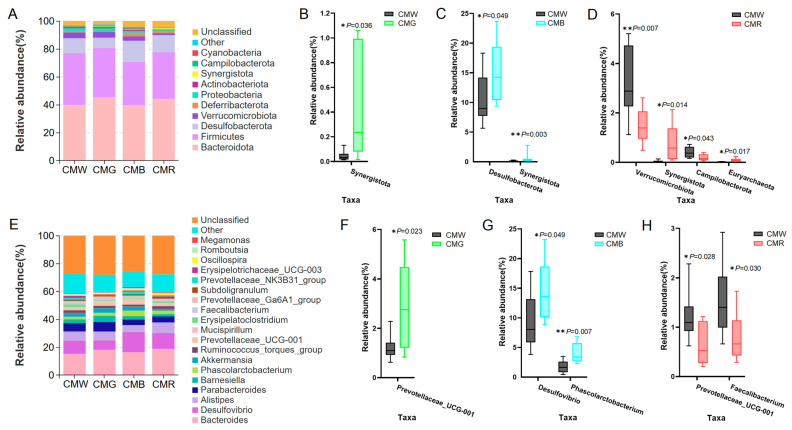
Distribution and difference analysis of cecal microbiota at both the phylum and genus levels. (**A**) Distribution of microbiota at the phylum level (top 10). (**B**–**D**) Taxonomic profiles of the notably significantly different bacteria at the phylum level when treated with green, blue, or red light in comparison with white light. (**E**) Stacked column chart depicting the top-19 microbiota distribution at the genus level (average relative abundance >0.5% in the WL, GL, BL, and RL). (**F**–**H**) Examination of significant differences of microbiota at the genus level under different light treatments: green light (**F**), blue light (**G**), and red light (**H**). Student’s *t*-test was used to calculate *p*-values. * represents *p* < 0.05, and ** represents *p* < 0.01. CMW, CMG, CMB, and CMR represent the cecal microbiota under white light, green light, blue light, and red light conditions, respectively.

The impact of monochromatic light on the gut microbial composition was also analyzed at both the phylum and genus levels. At the phylum level, the predominant bacterial groups in the cecum were *Bacteroidota* (CMW: 39.67%, CMG: 45.37%, CMB: 39.47%, and CMR: 44.18%), *Firmicutes* (CMW: 37.27%, CMG: 35.20%, CMB: 31.18%, and CMR: 33.48%), and *Desulfobacterota* (CMW: 10.63%, CMG: 7.33%, CMB: 15.05%, and CMR: 12.15%) (Figure 6A). Compared with the CMW group, the relative abundance of *Synergistota* was significantly elevated in the CMG, CMB, and CMR groups, *Desulfobacterota* showed a significant increase in the CMB group, and *Euryarchaeota* was notably higher in the CMR group (Figure 6B, C). Conversely, the relative abundances of *Verrucomicrobiota* and *Campilobacterota* in the CMR group were significantly lower (Figure 6D). In total, nineteen genera were identified that were present at a relative abundance greater than 0.5% in the WL, GL, BL, and RL (Figure 6E). Among the dominant bacterial genera, *Bacteroides* (CMW: 15.14%, CMG: 18.02%, CMB: 16.40%, and CMR: 18.65%), *Phascolarctobacterium* (CMW: 1.80%, CMG: 2.14%, CMB: 4.00%, and CMR: 2.50%), and *Desulfovibrio* (CMW: 9.50%, CMG: 6.79%, CMB: 14.52%, and CMR: 11.50%) were the most prevalent across the four groups (Figure 6E). Moreover, the relative abundance of the *Prevotellaceae UCG-001* was found to be significantly higher in the CMG group (Figure 6F), while *Desulfovibrio* and *Phascolarctobacterium* in the CMB group were more abundant when compared to the CMW group (Figure 6G). Additionally, the CMR group exhibited a decreased relative abundance of *Prevotellaceae UCG-001* and *Faecalibacterium*, in comparison with the CMW group (Figure 6H).

### 3.8. Linear Discriminant Analysis

**Figure 7:**
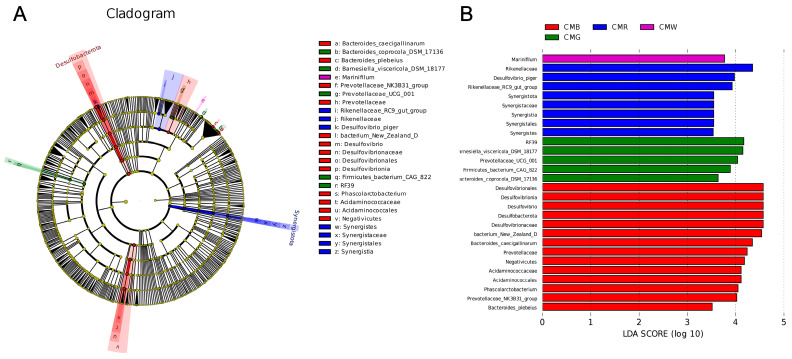
Analysis of cecal microflora differences under different light treatments by using the LEFse method. (**A**) Cladogram. (**B**) Histogram. LDA score (log10) = 3.5 as cutoff value. Blue, green, red, and purple represent different light treatment groups. CMW, CMG, CMB, and CMR represent the cecal microbiota under white light, green light, blue light, and red light conditions, respectively.

The linear discriminant analysis (LDA) effect size (LEfSe) method was used to identify microflora with significant abundance differences among the four groups. The cutoff value of the LDA score (log10) was 3.5. As illustrated in Figure 7, *Marinifilum* was significantly enriched in the CMW group. Moreover, *Rikenellaceae*, *Desulfovibrio piger*, and *Synergistota-Synergistes* (from phylum to genus level) were significantly enriched in the CMR group. In the CMG group, significant enrichment was observed for *Firmicutes bacterium CAG 822-RF39* (ranging from class to order), *Barnesiella viscericola DSM 18177*, *Prevotellaceae UCG 001*, and *Bacteroides coprocola DSM 17136*. Similarly, the CMB group exhibited a significant enrichment of *Bacteroides caecigallinarum*, *Bacteroides plebeius*, *Prevotellaceae NK3B31 group*, *Bacterium New Zealand D-Desulfovibrionia* (spanning from domain to genus), and *Phascolarctobacterium-Negativicutes* (spanning from phylum to family).

### 3.9. Correlation Analysis Between Growth Performance, Melatonin and Its Receptors, Gut Health, and Gut Microbiota

**Figure 8:**
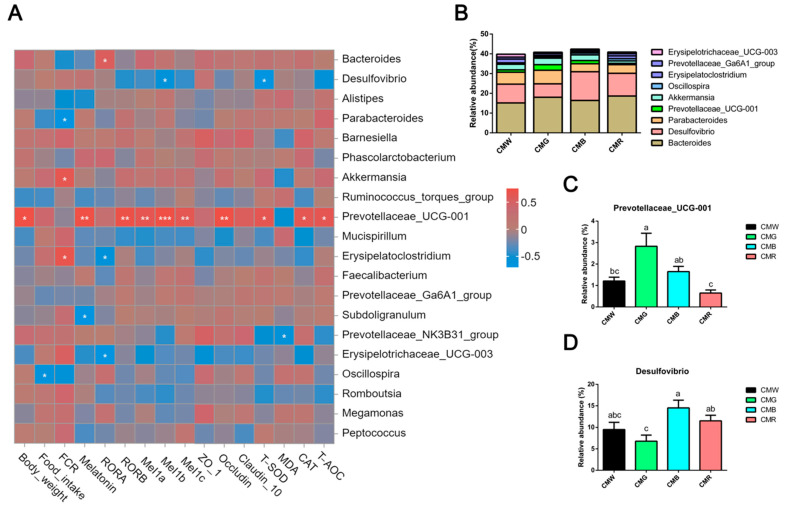
Spearman’s correlations between growth performance, melatonin levels, melatonin receptors gene expression, gut health, and microflora. (**A**) Spearman’s rank correlations analysis. The colors range from blue (negative correlations) to red (positive correlations). Statistical significance: * *p* < 0.05, ** *p* < 0.01, *** *p* < 0.001. (**B**) Genus-level histogram of relative abundance of cecal microorganisms that exhibited significant correlations. (**C**,**D**) Relative abundance of Prevotellaceae_UCG-001 (**C**) and Desulfovibrio (**D**) in different groups. One-way ANOVA was used to calculate *p*-values. Significant differences (*p* < 0.05) among WL, GL, BL, and RL groups are indicated by different superscript letters (a, b, c), while shared letters represent non-significant differences. CMW, CMG, CMB, and CMR represent the cecal microbiota under white light, green light, blue light, and red light conditions, respectively. ^a,b,c^ Different superscript letters indicate statistically significant differences (*p* < 0.05), while common letters denote non-significant differences.

To explore the specific bacteria associated with growth performance, melatonin and its receptors, and gut health (encompassing barrier and antioxidant functions), Spearman’s correlation analysis was conducted between the abundance of the top-20 microbiota and gut development. Monochromatic light affected approximately 40% of the total flora (Figure 8A,B). Moreover, there was a significant positive correlation (R = 0.55–0.80, *p* < 0.05) between body weight, melatonin, RORβ, Mel1a, Mel1b, Mel1c, occludin, T-SOD, CAT, T-AOC, and *Prevotellaceae_UCG-001* and a robust positive correlation between melatonin, its receptor, and *Prevotellaceae_UCG-001* (Figure 8A). Furthermore, *Prevotellaceae_UCG-001* was shown to be significantly more abundant in the green light environment than in the white and red light treatments (Figure 8C). Additionally, its relative abundance was significantly higher in the blue light treatment group than in the red light group. Another melatonin nuclear receptor, RORα, was significantly and positively correlated with *Bacteroides* and negatively correlated with *Erysipelatoclostridium* and *Erysipelotrichaceae_UCG-003*. In addition, significant positive correlations existed between *Akkermansia*, *Erysipelatoclostridium*, and FCR. Notably negative correlations between Mel1b, T-SOD, and *Desulfovibrio* were also identified from the heatmap. The relative abundance of *Desulfovibrio* in the intestines of the CMG group was significantly lower than that in the CMR and CMB groups (Figure 8D). The correlation analysis indicated that monochromatic light exposure could modulate gut health and microbial composition via the gut–microbiome–brain axis. Specifically, green light appeared to enhance the proliferation of beneficial bacteria, including *Prevotellaceae_UCG-001*, indicating a potential role in promoting intestinal homeostasis.

## 4. Discussion

As crucial environmental factors, light signals, particularly photoperiod, light color, and light intensity, have a significant impact on poultry production, behavior, and reproduction performance [21,22]. Past research has indicated that, among the various light colors, green light can enhance the body weight of poultry by stimulating their photoreceptors [23,24]. This study also confirmed that green light significantly boosted the body weight of 70-day-old Yangzhou geese, consistent with the findings reported by Xu et al. [24].

Light regulates growth and reproductive performance in poultry by regulating melatonin expression and receptor activity [25,26]. It has been reported that both green and blue light can significantly elevate serum melatonin levels in 14-day-old broilers compared to white or red light [27]. This finding aligns with the current observation that green light enhances melatonin secretion in the serum of 70-day-old geese. However, a minor effect of monochromatic light on serum melatonin levels was observed in 35-day-old broilers [28]. These discrepant results may be attributed to variations in growth stage, photoperiod, and light intensity among studies.

Melatonin exerts diverse physiological effects through distinct receptors [29,30]. The distribution and expression of melatonin receptors vary across different tissues and organs. Notably, in Yangzhou geese, the Mel1a receptor is highly expressed in the granulosa cells, ovaries, and small intestine tissues, whereas, in chickens, only the Mel1b and Mel1c receptors are expressed in the granulosa cell layer [31,32]. This study identified the presence of both membrane-bound and nuclear melatonin receptors in jejunal tissues. Several of these receptors exhibit distinct functions. For instance, Mel1a has been identified as a potential factor that influences egg production in Yangzhou geese, whereas melatonin has been shown to facilitate spleen development and immune cell expansion in broiler chickens through Mel1b and Mel1c receptors [33,34]. Moreover, the expression of melatonin receptors is modulated by monochromatic light, which subsequently affects the physiological parameters in animals. Specifically, green light has been shown to enhance the expression of membrane receptors while reducing nuclear receptor expression in the spleen of chickens compared to red light and is associated with an increase in T/B lymphocyte populations [34]. This study demonstrated that the expression levels of melatonin receptors were higher under green and blue light than under other light colors. These findings align with those in previous studies on broiler embryos, which indicated that green light enhances the expression of jejunal Mel1a, Mel1b, and Mel1c receptors [35].

Alterations in the intestinal melatonin receptors induced by light have a direct impact on the structure and function of the gut. First, light color modulates the intestinal villi morphology within the goose, which correlates with the digestive and absorptive capabilities of poultry. Specifically, green light was observed to enhance both VH and VH/CD of duodenum, jejunum, and ileum, a finding that aligns with the results reported by Xie et al. [36]. Second, green light significantly upregulates the mRNA expression levels of jejunal tight junction proteins in geese and broilers [16]. Third, the reductive capacity of the cecum was improved by green or blue light exposure, as the activities of jejunal T-SOD, T-AOC, and CAT were elevated under green or blue light conditions, which is consistent with previous reports [16,37]. The influence of light color on the structure and function of the gut is mediated by melatonin and its receptors, which is supported by the observation that the removal of the pineal gland attenuates the effects of light color on the gut [38,39,40,41]. In summary, green light improved intestinal health by enhancing intestinal absorption and strengthening the intestinal barrier.

In addition to directly influencing intestinal structure and function, monochromatic light indirectly affects the diversity of microflora within intestinal commensals [16,42]. The current study revealed that *Firmicutes* and *Bacteroidetes* at the phylum level, along with *Bacteroides* at the genus level, represented the predominant active bacteria, consistent with previous observations in Yangzhou geese and broilers [24,43]. Moreover, an increased abundance of *Bacteroides* and *Prevotellaceae* was observed in the cecum of green light-exposed Yangzhou geese. Both *Bacteroides* and *Prevotellaceae* are enriched in the cecum of Yangzhou geese with high body weights [24], and an elevated relative abundance of *Bacteroides* improves the growth performance of Zi geese by augmenting antioxidant capacity, improving gut morphology, and altering cecal microbial composition [44]. These observations indicate that the improvement in growth performance by green light is due to the regulation of the composition of the intestinal flora, particularly by increasing the abundance of *Bacteroides* and *Prevotellaceae*. Correlation analyses of growth performance, melatonin levels, and intestinal function with gut microbiota composition further support this relationship. Notably, the *Prevotellaceae_UCG-001* strain, a member of the *Prevotellaceae* family, exhibited significant positive correlations with body weight, melatonin, and its receptor expression, as well as intestinal health markers. The *Prevotellaceae_UCG_001* strain enhances the levels of medium- and short-chain fatty acids within the gut, which are linked to antioxidant capacity and immune function [45]. This strain also facilitates the growth of broilers and augments egg production in laying hens [46,47]. Therefore, the positive association of *Prevotellaceae_UCG_001* with body weight and gut function indicators, along with its growth-promoting effects, indicates that light modulates gut health and microbial composition via melatonin and its receptors, consequently affecting animal growth.

## 5. Conclusions

In conclusion, green light exposure exerted multiple beneficial effects in geese, including (1) elevation of serum melatonin concentrations, (2) upregulation of intestinal melatonin receptor gene expression, (3) improvement of intestinal morphology (increased villus height-to-crypt depth ratio), (4) enhancement of antioxidant capacity, and (5) modulation of gut microbial composition. These coordinated physiological responses collectively contributed to the observed increase in the body weight of geese.

## Figures and Tables

**Table 1:** Primers used in this study.

Genes	Primer Sequence (5′ to 3′)	Accession Number
Mella-F	GTTCCGCAGCTTCTTGTTG	XM_066996266.1
Mella-R	CTGGGTCACCTCCACCTTG	
Mel1b-F	TGTGGTAATTCATTTCATCGTCCC	U30609
Mel1b-R	TTGGTGCCATTTCTGAAGGATTGAT	
Mel1c-F	CAGATAAGTGGGTTCCTGATGGG	U31821
Mel1c-R	ACCGAAGGCTGTGGCAGATGTAG	
RORα-F	GGCAGTTATGCGCAGTCAAA	XM_067003930.1
RORα-R	TTCTGGGAGTCAAAGGCACG	
RORβ-F	GCAATGGCTTGAGCAACCTG	XM_048073568.1
RORβ-R	GCTGGGCAGAATCCACATTG	
GAPDH-F	GCCATCACAGCCACACAGA	XM_067004670.1
GAPDH-R	TTTTCCCACAGCCTTAGCA	
ZO-1-F	TACGCTGTTGAATGTCCC	XM_013177403.1
ZO-1-R	ATGGTCTGAAGGCTCTGA	
occludin-F	TCCCGCCGCTTCTACCT	XM_013199669.1
occludin-R	CACCTGGCTGCACATGG	
claudin-10F	ATGACTGGTTGTTCCCTGTA	XM_013201623.1
claudin-10R	AGCCCATCCAATGAATAAAG	

**Table 2:** Effects of monochromatic light on growth performance of Yangzhou geese.

Items	Treatments ^1^				SEM	*p*-Value
	White Light	Green Light	Blue Light	Red Light		
1d BW(g)	98.47	97.86	97.76	97.35	1.82	0.9386
70d BW(g)	4336 ^bc^	4537 ^a^	4425 ^abc^	4254 ^c^	0.35	0.0013
ADFI(g)	200.46 ^a^	199.68 ^a^	198.93 ^a^	187.69 ^b^	4.54	0.0486
FCR	3.05	3.01	3.08	2.98	0.09	0.6616

^1^ Data represent the means of four replicates. BW: Body weight; ADFI: Average daily feed intake; FCR (Feed conversion ratio) = Total feed consumption/Total weight gain. ^a,b,c^ Different superscript letters indicate statistically significant differences (*p* < 0.05), while shared letters denote non-significant differences.

**Table 3:** The effect of different monochromatic light on intestinal morphology of Yangzhou geese.

Items ^1^	Treatments ^1^				SEM	*p*-Value
	White Light	Green Light	Blue Light	Red Light		
Duodenum						
VH, μm	3339.20 ^c^	4645.45 ^a^	4299.88 ^a^	3371.95 ^c^	134.81	<0.0001
CD, μm	1401.03 ^b^	1007.49 ^c^	973.92 ^c^	1527.66 ^a^	52.47	
VH/CD	2.39 ^b^	4.62 ^a^	4.45 ^a^	2.22 ^b^	0.17	
Jejunum						
VH, μm	3869.17 ^c^	4822.63 ^a^	4263.07 ^b^	3271.82 ^d^	82.48	<0.0001
CD, μm	1250.97 ^b^	852.91 ^d^	1098.46 ^c^	1438.09 ^a^	52.79	
VH/CD	3.11 ^c^	5.68 ^a^	3.9 ^b^	2.29 ^d^	0.19	
Ileum						
VH, μm	3329.6 ^b^	4092 ^a^	3931.18 ^a^	2972.76 ^c^	120.31	<0.0001
CD, μm	1000.18 ^b^	773.44 ^c^	819.52 ^c^	1297.44 ^a^	34.41	
VH/CD	3.36 ^b^	5.3 ^a^	4.83 ^a^	2.29 ^c^	0.24	

^1^ Data represent the means of six samples. VH: Villus height; CD: Crypt depth; VH/CD: Villus height/Crypt depth. SEM: Standard error of the mean. ^a,b,c,d^ Different superscript letters indicate statistically significant differences (*p* < 0.05), while common letters denote non-significant differences.

## Data Availability

The raw sequencing data were uploaded to the Genome Sequence Archive (accession number: PRJCA029767). All data generated or analyzed during this study are included in this published article.

## Abbreviations

The following abbreviations are used in this manuscript:

ADFI	average daily feed intake
BL	blue light
BW	body weight
CAT	catalase
CD	crypt depths
CMB	cecal microflora in the blue light group
CMG	cecal microflora in the green light group
CMR	cecal microflora in the red light group
CMW	cecal microflora in the white light group
FCR	feed conversion ratio
GAPDH	glyceraldehyde-3-phosphate dehydrogenase
GL	green light
H&E	hematoxylin and eosin staining
MDA	malondialdehyde
Mel	melatonin membrane receptor
LDA	linear discriminant analysis
LEfSe	linear discriminant analysis effect size
OTUs	operational taxonomic units
qRT-PCR	quantitative real-time polymerase chain reaction
RL	red light
ROR	melatonin nuclear receptors
RORα	retinoic acid receptor-related orphan receptor α
RORβ	retinoic acid receptor-related orphan receptor β
T-AOC	total antioxidant capacity
T-SOD	total superoxide dismutase
VH	villus heights
WL	white light
ZO-1	zonula occludens-1

## References

[1] Prayitno D., Phillips C., Omed H. (1997). The effects of color of lighting on the behavior and production of meat chickens. Poult. Sci..

[2] Zhu H., Hu M., Guo B., Qu X., Lei M., Chen R., Chen Z., Shi Z. (2019). Effect and molecular regulatory mechanism of monochromatic light colors on the egg-laying performance of Yangzhou geese. Anim. Reprod. Sci..

[3] Franco B.R., Shynkaruk T., Crowe T., Fancher B., French N., Gillingham S., Schwean-Lardner K. (2022). Light color and the commercial broiler: Effect on behavior, fear, and stress. Poult. Sci..

[4] Hunt D.M., Carvalho L.S., Cowing J.A., Davies W.L. (2009). Evolution and spectral tuning of visual pigments in birds and mammals. Philos. Trans. R. Soc. Lond. B Biol. Sci..

[5] Hart N.S. (2001). The Visual Ecology of Avian Photoreceptors. Prog. Retin. Eye Res..

[6] Yang Y., Pan C., Zhong R., Pan J. (2018). The quantitative models for broiler chicken response to monochromatic, combined, and mixed light-emitting diode light: A meta-analysis. Poult. Sci..

[7] Wang P., Sun Y., Li Y., Fan J., Zong Y., Isa A.M., Shi L., Wang Y., Ni A., Ge P. (2021). Monochromatic green light stimulation during incubation shortened the hatching time via pineal function in White Leghorn eggs. J. Anim. Sci. Biotechnol..

[8] Chen Z., Qu X., Feng C., Guo B., Zhu H., Yan L. (2022). Monochromatic Green Light Stimulation during Incubation Alters Hepatic Glucose Metabolism That Improves Embryonic Development in Yangzhou Goose Eggs. Int. J. Mol. Sci..

[9] Xu X., Fan S., Wu H., Li H., Shan X., Wang M., Zhang Y., Xu Q., Chen G. (2024). A 16S RNA Analysis of Yangzhou Geese with Varying Body Weights: Gut Microbial Difference and Its Correlation with Body Weight Parameters. Animals.

[10] Hao C., Wu D., Mo L., Xu G. (2024). A review on gut microbial diversity and function of overwintering animals. Biodivers. Sci..

[11] Liu G., Luo X., Zhao X., Zhang A., Jiang N., Yang L., Huang M., Xu L., Ding L., Li M. (2018). Gut microbiota correlates with fiber and apparent nutrients digestion in goose. Poult. Sci..

[12] Wei R., Ye F., He F., Song Q., Xiong X., Yang W., Gang X., Hu J., Hu B., Xu H. (2021). Comparison of overfeeding effects on gut physiology and microbiota in two goose breeds. Poult. Sci..

[13] Li G., Wang X., Liu Y., Wang C., Yang Y., Gong S., Zhu L., He D., Wang H. (2022). Supplementation with honeysuckle extract improves growth performance, immune performance, gut morphology, and cecal microbes in geese. Front. Vet. Sci..

[14] Zhang X., Akhtar M., Chen Y., Ma Z., Liang Y., Shi D., Cheng R., Cui L., Hu Y., Nafady A.A. (2022). Chicken jejunal microbiota improves growth performance by mitigating intestinal inflammation. Microbiome.

[15] Zhang X., Hu Y., Ansari A.R., Akhtar M., Chen Y., Cheng R., Cui L., Nafady A.A., Elokil A.A., Abdel-Kafy E.M. (2021). Caecal microbiota could effectively increase chicken growth performance by regulating fat metabolism. Microb. Biotechnol..

[16] Zhang Y., Wang Z., Dong Y., Cao J., Chen Y. (2022). Blue Light Alters the Composition of the Jejunal Microbiota and Promotes the Development of the Small Intestine by Reducing Oxidative Stress. Antioxidants.

[17] Carrillo-Vico A., Lardone P.J., Álvarez-Sánchez N., Rodríguez-Rodríguez A., Guerrero J.M. (2013). Melatonin: Buffering the immune system. Int. J. Mol. Sci..

[18] Zhang H., Zhang Y. (2014). Melatonin: A well-documented antioxidant with conditional pro-oxidant actions. J. Pineal Res..

[19] Kankova Z., Drozdova A., Hodova V., Zeman M. (2022). Effect of blue and red monochromatic light during incubation on the early post-embryonic development of immune responses in broiler chicken. Br. Poult. Sci..

[20] Nguyen T.N.D., Le H.N., Eva P., Alberto F., Le T.H. (2021). Relationship between the ratio of villous height: Crypt depth and gut bacteria counts aswell production parameters in broiler chickens. J. Agric. Dev..

[21] Barros J.S.G., Sartor K., Pedroso T.F., Vasconcelos H., Scopacasa V.A., Bottura J.R., Sena R.G., Salvador M.J., de Moura D.J. (2024). Impact of light spectrum electromagnetic radiation variations on performance and hormonal profiles in laying hens. Sci. Rep..

[22] Pan C., Xiang R., Pan J. (2024). Lighting quality evaluation on growth performance and feather pecking behavior of broilers. Poult. Sci..

[23] Oke O.E., Oni A.I., Adebambo P.O., Oso O.M., Adeoye M.M., Lawal T.G., Afolayan T.R., Ogunbajo O.E., Ojelade D.I., Bakre O.A. (2020). Evaluation of light colour manipulation on physiological response and growth performance of broiler chickens. Trop. Anim. Health Prod..

[24] Xu Y., Tang Y., Cheng Y., Yang W., Liu J., Guo B., Luo G., Zhu H. (2025). Effects of different monochromatic light on growth performance and liver circadian rhythm of Yangzhou geese. Poult. Sci..

[25] Liu X., Wang L., Wang Z., Dong Y., Chen Y., Cao J. (2021). Mel1b and Mel1c melatonin receptors mediate green light-induced secretion of growth hormone in chick adenohypophysis cells via the AC/PKA and ERK1/2 signalling pathways. J. Photochem. Photobiol. B.

[26] Bao Q., Gu W., Song L., Weng K., Cao Z., Zhang Y., Zhang Y., Ji T., Xu Q., Chen G. (2023). The Photoperiod-Driven Cyclical Secretion of Pineal Melatonin Regulates Seasonal Reproduction in Geese (*Anser cygnoides*). Int. J. Mol. Sci..

[27] Jin E., Jia L., Li J., Yang G., Wang Z., Cao J., Chen Y. (2011). Effect of monochromatic light on melatonin secretion and arylalkylamine *N*-acetyltransferase mRNA expression in the retina and pineal gland of broilers. Anat. Rec..

[28] Franco B.R., Shynkaruk T., Crowe T., Fancher B., French N., Gillingham S., Schwean-Lardner K. (2022). Light wavelength and its impact on broiler health. Poult. Sci..

[29] Xiong J., Wang Z., Dong Y., Cao J., Chen Y. (2024). The signal pathway of melatonin mediates the monochromatic light-induced T-lymphocyte apoptosis in chicken thymus. Poult. Sci..

[30] Xiong J., Wang Z., Dong Y., Cao J., Chen Y. (2024). Melatonin nuclear receptors mediate monochromatic light-induced T-lymphocyte proliferation of thymus through the AKT/GSK3β/β-catenin pathway in chick. Poult. Sci..

[31] Sundaresan N.R., Leo M.D.M., Subramani J., Anish D., Sudhagar M., Ahmed K.A., Saxena M., Tyagi J.S., Sastry K.V.H., Saxena V.K. (2009). Expression analysis of melatonin receptor subtypes in the ovary of domestic chicken. Vet. Res. Commun..

[32] Alsiddig M.A., Yu S.G., Pan Z.X., Widaa H., Badri T.M., Chen J., Liu H.L. (2017). Association of single nucleotide polymorphism in *melatonin receptor 1A* gene with egg production traits in Yangzhou geese. Anim. Genet..

[33] Guo Q., Dong Y., Cao J., Wang Z., Zhang Z., Chen Y. (2015). Developmental changes of melatonin receptor expression in the spleen of the chicken, Gallus domesticus. Acta Histochem..

[34] Xiong J., Wang Z., Cao J., Dong Y., Chen Y. (2020). Melatonin mediates monochromatic light–induced proliferation of T/B lymphocytes in the spleen via the membrane receptor or nuclear receptor. Poult. Sci..

[35] Yu Y. (2014). Effects of Different Light Wavelengths on Development of Small Intestine and Bursa in Chick Embryos during the Late Period and Its Modulation Mechanisms.

[36] Xie D., Li J., Wang Z.X., Cao J., Li T.T., Chen J.L., Chen Y.X. (2011). Effects of monochromatic light on mucosal mechanical and immunological barriers in the small intestine of broilers. Poult. Sci..

[37] Li J., Cao J., Wang Z., Dong Y., Chen Y. (2015). Melatonin plays a critical role in inducing B lymphocyte proliferation of the bursa of Fabricius in broilers via monochromatic lights. J. Photochem. Photobiol. B Biol..

[38] Cui Y.-M., Wang J., Zhang H.-J., Qi G.-H., Qiao H.-Z., Gan L.-P., Wu S.-G. (2022). Effect of Changes in Photoperiods on Melatonin Expression and Gut Health Parameters in Laying Ducks. Front. Microbiol..

[39] Zhang Y., Wang Z., Dong Y., Cao J., Chen Y. (2022). Effects of Different Monochromatic Light Combinations on Cecal Microbiota Composition and Cecal Tonsil T Lymphocyte Proliferation. Front. Immunol..

[40] Liu Y., He Y., Fan S., Gong X., Zhou Y., Jian Y., Ouyang J., Jiang Q., Zhang P. (2023). Effects of LED Light Colors on the Growth Performance, Intestinal Morphology, Cecal Short-Chain Fatty Acid Concentrations and Microbiota in Broilers. Animals.

[41] Zheng J., Zhou Y., Zhang D., Ma K., Gong Y., Luo X., Liu J., Cui S. (2024). Intestinal melatonin levels and gut microbiota homeostasis are independent of the pineal gland in pigs. Front. Microbiol..

[42] Li X., Zheng Z., Pan J., Jiang D., Tian Y., Fang L., Huang Y. (2020). Impacts of colored light-emitting diode illumination on the growth performance and fecal microbiota in goose. Poult. Sci..

[43] Wei S., Morrison M., Yu Z. (2013). Bacterial census of poultry intestinal microbiome. Poult. Sci..

[44] Cui Y., Leng X., Zhao Y., Zhao Y., Wang Q. (2024). Effects of dietary Artemisia annua supplementation on growth performance, antioxidant capacity, immune function, and gut microbiota of geese. Poult. Sci..

[45] Moore K., Pearston F., Pritchard T., Wall E., Coffey M., Nani J., Mucha S., McLaren A., Mrode R., Conington J. (2021). Effects of supplementing sow diets during late gestation with Pennisetum purpureum on antioxidant indices, immune parameters and faecal microbiota. Vet. Med. Sci..

[46] Qi Z., Shi S., Tu J., Li S. (2019). Comparative metagenomic sequencing analysis of cecum microbiotal diversity and function in broilers and layers. 3 Biotech.

[47] Wang X., Wu X., Cong X., Ren J., Li J., Zhu J., Dai M., Hrabchenko N., Du Y., Qi J. (2022). The functional role of fecal microbiota transplantation on Salmonella Enteritidis infection in chicks. Vet. Microbiol..

